# Evidence-informed health policy 1 – Synthesis of findings from a multi-method study of organizations that support the use of research evidence

**DOI:** 10.1186/1748-5908-3-53

**Published:** 2008-12-17

**Authors:** John N Lavis, Andrew D Oxman, Ray Moynihan, Elizabeth J Paulsen

**Affiliations:** 1 Centre for Health Economics and Policy Analysis, Department of Clinical Epidemiology and Biostatistics, McMaster University, 1200 Main St. West, HSC-2D3, Hamilton, ON L8N 3Z5, Canada; 2 Department of Political Science, McMaster University, 1200 Main St. West, HSC-2D3, Hamilton, ON L8N 3Z5, Canada; 3 Norwegian Knowledge Centre for the Health Services, Pb. 7004, St. Olavs plass, Oslo N-0130, Norway; 4 School of Medicine and Public Health, Faculty of Health, The University of Newcastle, Medical Sciences Building – Level 6, Callaghan, NSW 2308, Australia

## Abstract

**Background:**

Organizations have been established in many countries and internationally to support the use of research evidence by producing clinical practice guidelines, undertaking health technology assessments, and/or directly supporting the use of research evidence in developing health policy on an international, national, and state or provincial level. Learning from these organizations can reduce the need to 'reinvent the wheel' and inform decisions about how best to organize support for such organizations, particularly in low- and middle-income countries (LMICs).

**Methods:**

We undertook a multi-method study in three phases – a survey, interviews, and case descriptions that drew on site visits – and in each of the second and third phases we focused on a purposive sample of those involved in the previous phase. We used the seven main recommendations that emerged from the advice offered in the interviews to organize much of the synthesis of findings across phases and methods. We used a constant comparative method to identify themes from across phases and methods.

**Results:**

Seven recommendations emerged for those involved in establishing or leading organizations that support the use of research evidence in developing health policy: 1) collaborate with other organizations; 2) establish strong links with policymakers and involve stakeholders in the work; 3) be independent and manage conflicts of interest among those involved in the work; 4) build capacity among those working in the organization; 5) use good methods and be transparent in the work; 6) start small, have a clear audience and scope, and address important questions; and 7) be attentive to implementation considerations, even if implementation is not a remit. Four recommendations emerged for the World Health Organization (WHO) and other international organizations and networks: 1) support collaborations among organizations; 2) support local adaptation efforts; 3) mobilize support; and 4) create global public goods.

**Conclusion:**

This synthesis of findings from a multi-method study, along with the more detailed findings from each of the three phases of the study (which are reported in the three following articles in the series), provide a strong basis on which researchers, policymakers, international organizations (and networks) like WHO can respond to the growing chorus of voices calling for efforts to support the use of research evidence in developing health policy.

## Background

Over the past three years, there has been a great deal of international discussion about how to harness health research evidence more effectively in order to achieve the United Nations' millennium development goals as well as national health goals, particularly in low- and middle-income countries (LMICs). One important focus in this discussion has been the call to develop mechanisms to support the use of research evidence in developing clinical practice guidelines, health technology assessments, and health policy. The chapter on linking research to action in the World Report on Knowledge for Better Health that was released by the World Health Organization (WHO) in early November 2004 provided a framework for appreciating the diversity and complementarities of many of these mechanisms [1]. The health ministers and heads of national delegations from 58 countries who participated in the Ministerial Summit on Health Research that was held in Mexico City in November 2004 reiterated the call for developing such support mechanisms [2].

At the World Health Assembly that was held in Geneva in May 2005, these debates culminated in the passage of a two-part resolution that established specific accountabilities for developing mechanisms to support the use of research evidence in developing health policy [3]. The first part of the resolution called on WHO member states to 'establish or strengthen mechanisms to transfer knowledge in support of evidence-based public health and health-care delivery systems, and evidence-based health-related policies.' The second part of the resolution called on WHO's Director-General to 'assist in the development of more effective mechanisms to bridge the divide between ways in which knowledge is generated and ways in which it is used, including the transformation of health-research findings into policy and practice.'

Organizations have already been established in many countries and internationally to support the use of research evidence in developing health policy. These include organizations that produce clinical practice guidelines (CPG), undertake health technology assessment (HTA), and directly support the use of research evidence in developing health policy on an international, national, and state or provincial level (hereafter called government support units, or GSUs). While there may be important differences among these organizations, there may also be many commonalities and opportunities for existing and new organizations to learn from this collective experience. A review of this experience can reduce the need to 'reinvent the wheel' and inform decisions about how best to organize support for evidence-informed health policy development processes.

An important dimension of the context for any such review is the growing demand for more rigorous processes to ensure that decision-making is well informed by the best available research evidence. These processes, in contrast with traditional approaches that rely heavily on the opinions of experts, demand systematic and transparent approaches to access, synthesise, and interpret research evidence and to integrate that evidence with other information, values, and judgements in order to formulate recommendations or make decisions. The need for more rigorous processes to support clinical decision-making is underscored by evidence of inconsistencies between the available research evidence and expert recommendations [4,5], insufficient use of the available research evidence [6,7], and other shortcomings in how guidelines and recommendations are developed [8-15]. Similar criticisms have been voiced and calls made for the better use of research evidence in health systems management and public policy-making [1-3,16-18].

Our objective was to identify organizations around the world, and especially in LMICs, that are in some way successful or innovative in supporting the use of research evidence in the development of CPGs, HTAs, and health policy, and to describe their experiences. We pursued this objective in a three-phase, multi-method study [19]. In this article, we provide a synthesis of findings from across phases and methods. In the following three articles in the series, we provide more detail about the methods and findings from each of the three phases (Table 1).

**Table 1:** Overview of the four-article series

**This article**	**Synthesis of findings from the three-phase, multi-method study**
[20]	Survey of a senior staff member (the director or his or her nominee) of clinical practice guideline-producing organizations, HTA agencies, and government support units

[21]	Interview with the senior staff member of a purposively sampled sub-group of these three types of organizations, with an emphasis on those organizations that were particularly successful or innovative

[22]	Case descriptions (based on site visits) of one or more organizations supporting the use of research evidence from among the cases described in the interviews and (once) other cases with which we were familiar, again with an emphasis on those organizations that were particularly successful or innovative

## Methods

In order to support our primary focus on LMICs, we convened a project reference group that drew on two or three individuals who were from, or who are very knowledgeable about, Africa, Asia, or Latin America, as well as individuals from North America, Europe (including a representative from the project funder), and WHO (including members of the Advisory Committee on Health Research). Collectively, the reference group provided many perspectives on the three types of organizations under study and on potential country- or region-level differences in the opportunities and challenges confronting these organizations. The reference group provided feedback on our draft protocol, study population, questionnaire, interview guide, and case study data collection plan. We also engaged one individual from each of Africa, Asia, Europe, and Latin America to provide a very detailed review of the draft final report on which this series of articles is based.

We undertook the project in three phases – a survey, interviews, and case descriptions that drew on site visits – and in each of the second and third phases we focused on a purposive sample of those involved in the previous phase. We drew on many people and organizations around the world to generate a list of organizations to survey. We modified a questionnaire that had been developed originally by the Appraisal of Guidelines, Research and Evaluation in Europe (AGREE) collaboration, adapted one version of the questionnaire for organizations producing CPGs and HTAs and another for GSUs, piloted both versions of the questionnaire, and made a small number of final modifications to both versions of the questionnaire [20]. We sent the questionnaire by email to 176 organizations and followed up periodically with non-responders by email and telephone. We then purposively sampled 25 organizations from among those who responded to the survey. We developed and piloted an interview guide and used the guide to conduct interviews by telephone with the director of each organization [21]. We then purposively sampled eight cases of one or more organizations supporting the use of research evidence from among the cases described in the interviews and (once) from among other cases with which we were familiar. We developed and piloted a case study data collection plan and conducted site visits for each case [22]. Data collection for the case studies included interviews with 51 key informants and a review of publicly available documents. We conducted simple descriptive statistics using the quantitative survey data and we analysed the written survey responses, interviews, in-person interviews, and documents using a constant comparative method of analysis. We produced a video documentary about each case.

We used the seven main recommendations that emerged from the advice offered in the interviews to organize much of the synthesis of findings across phases and methods. We chose this organizing framework for three reasons: 1) our interest is in the views and experiences of particularly successful or innovative groups (particularly those based in LMICs), and the interviews allowed us to balance breadth and depth in soliciting these views and experiences; 2) the thematic analysis of the interview data yielded clear recommendations for other organizations; and 3) the analysis of the quantitative survey data, the written survey responses (which we call the qualitative survey data), and the case descriptions reinforced the broad applicability of the organizing framework. We used a constant comparative method to identify from across phases and methods themes relevant to WHO and other international organizations and networks.

The principal investigator for the overall project (AO), who is based in Norway, confirmed that, in accordance with the country's act on ethics and integrity in research, this study did not require ethics approval from one of the country's four Regional Committees for Medical and Health Research Ethics.

## Results

Seven recommendations emerged from the multi-method study for those involved in establishing or leading organizations that support the use of research evidence in developing health policy:

### Collaborate with other organizations

This advice was reinforced by: 1) the (quantitative) survey finding that more than half of the organizations (and particularly HTA agencies) reported that examples from other countries were helpful in establishing their organization; 2) the (qualitative) survey finding that many organizations producing CPGs or HTAs conducted a focused review of one particular organization that they then emulated or a broad review of a variety of organizational models; 3) the (qualitative) survey finding that the advice that was most commonly offered by organizations producing CPGs, HTAs, or both was to seek support from similar existing organizations or networks, whether through informal interactions, study tours, mentoring relationships, twinning, partnerships or network memberships; 4) the (qualitative) survey finding that working within national networks and, more generally, collaborating rather than competing with other bodies, was a commonly cited strength in how GSUs are organized; and 5) the case descriptions finding that one of the two types of advice offered to other organizations was to learn from other organizations.

### Establish strong links with policymakers and involve stakeholders in the work

This advice was reinforced by: 1) the (quantitative) survey finding that a high proportion (88%) of GSUs involved target users in the selection of topics or the services undertaken; 2) the interview finding that, while informal relationships with policymakers were identified more frequently as important by GSUs than by organizations producing CPGs, HTAs, or both, nearly all of the organizations reported using personal communications with decision-makers, and particularly with policymakers; 3) the interview finding that organizations both within and outside government viewed their close links with policymakers as a strength; and 4) the case descriptions finding that the existence of a strong relationship between researchers and policymakers was repeatedly cited as one of two key organizational strengths (although this strength brought with it a related challenge, namely the need to manage the conflicts of interest that can emerge in any close relationship between researchers and policymakers).

### Be independent and manage conflicts of interest among those involved in the work

This advice was reinforced by: 1) the (qualitative) survey finding that independence is by far the most commonly cited strength in how organizations producing CPGs and HTAs are organized; and 2) the case descriptions finding that the presence of conflicts of interest was repeatedly cited as one of two key organizational weaknesses.

### Build capacity among those working in the organization

This advice was reinforced by: 1) the (quantitative) survey finding that most organizations have a small number of full-time equivalent (FTE) staff; 2) and the case descriptions finding that developing capacity among and retaining skilled staff and collaborators was one of their two frequently offered types of advice.

### Use good methods and be transparent in the work

This advice was reinforced by: 1) the (quantitative) survey finding that between 84% and 100% of organizations reported providing panels with or using systematic reviews; 2) the (qualitative) survey finding that an evidence-based approach is the most commonly cited strength of the methods used by organizations that produce CPGs and HTAs; 3) the interview finding that using rigorous methods that are systematic and transparent (sometimes shortened to 'being evidence-based') was the most commonly cited strength among all organizations; and 4) the case descriptions finding that the use of an evidence-based approach was one of two organizational strengths that were repeatedly cited. However, all but one of the organizations producing CPGs, HTAs, or both used informal methods for setting priorities. Relatively few organizations producing CPGs and HTAs convened groups to develop CPGs or HTAs, took equity considerations into account, or had established a process for addressing conflicts of interest. GSUs were less likely to have a manual that described the methods they use and to conduct or use systematic reviews and more likely to report using non-systematic methods to review the literature. In addition, using systematic and transparent methods brought with it a related challenge, namely the time-consuming nature of an evidence-based approach.

### Start small, have a clear audience and scope, and address important questions

This finding was reinforced by: 1) the (qualitative) survey finding that the most commonly cited weakness in how these organizations are organized is a lack of resources, both financial and human; 2) the (qualitative) survey finding that the most commonly cited weakness of the methods used by organizations that produce CPGs and HTAs was their time-consuming and labour-intensive nature; 3) the (qualitative) survey finding that GSUs advised others establishing a similar organization to attend to the need for secure funding; 4) the interview finding that the weakness noted by most of the CPG- and HTA-producing organizations was inadequate resources, more specifically insufficient numbers of skilled staff and time, together with using labour- and time-intensive processes that limit the number and quality of CPGs and HTAs that can be produced and updated; and 5) the case descriptions finding that a lack of resources was repeatedly cited as one of two organizational weaknesses.

### Be attentive to implementation considerations even if implementation is not a remit

This advice was reinforced by: 1) the (quantitative) survey finding that less than half of all organizations provided a summary of take-home messages in their products; 2) the (quantitative) survey finding that between one-half and two-thirds of organizations do not collect data systematically about uptake; 3) the (qualitative) survey finding that the most commonly cited weaknesses of CPG- and HTA-producing organizations' outputs are the lack of dissemination and implementation strategies for the outputs, and the lack of monitoring and evaluation of impact; 4) the interview finding that most organizations argued that it is the clients who requested a CPG or HTA – typically, the minister of health or more generally the department of health – who is responsible for implementing recommendations or policy decisions; 5) the interview finding that all types of organizations tended to focus largely on weaknesses in implementation when asked about both strengths and weaknesses, with few exceptions; and 6) the interview finding that most of the examples of success among organizations producing CPGs, HTAs, or both were occasions where there was a perception that clinicians adhered to the organization's recommendations or policymakers based their decisions (at least in part) on the work of the organization.

Four recommendations emerged from the multi-method study for WHO, most of which were equally relevant for other international organizations and for networks such as the Guidelines International Network and the International Network of Agencies for Health Technology Assessment. The first recommendation was to support collaborations among organizations. This advice is supported by: 1) the (qualitative) survey finding that many CPG- and HTA-producing organizations argued that WHO should play a facilitating role in coordination efforts, primarily to avoid duplication; and 2) the interview finding that when comments about WHO's potential role were offered they almost always pertained to the need to foster collaborations across organizations. The second recommendation was to support local adaptation efforts. This advice is supported by: 1) the (qualitative) survey finding that some CPG- and HTA-producing organizations argued that WHO should play a facilitating role in local adaptation efforts in order to enhance local applicability; and 2) the (qualitative) survey finding that some GSUs argued that WHO should play a role in helping to adapt global evidence to local contexts or at least in supporting such processes. The third recommendation was to mobilize support. This advice is supported by the case descriptions finding that one of only two suggestions that were offered with any frequency was that WHO should play a role in mobilizing one or more of government support, financial resources, and the participation of both policymakers and researchers. And the fourth recommendation was to create global public goods, which was supported by the case description finding that the second of only two suggestions that were offered with any frequency was that WHO should play a role in creating knowledge-related global public goods.

## Discussion

### Principal findings from the multi-method study

By drawing on three phases of data collection and multiple methods, we identified seven recommendations for those involved in establishing or leading organizations that support the use of research evidence in developing health policy, particularly in LMICs: 1) collaborate with other organizations; 2) establish strong links with policymakers and involve stakeholders in the work; 3) be independent and manage conflicts of interest among those involved in the work; 4) build capacity among those working in the organization; 5) use good methods and be transparent in the work; 6) start small, have a clear audience and scope, and address important questions; and 7) be attentive to implementation considerations even if implementation is not a remit. We also identified four recommendations for WHO and other international organizations and networks: 1) support collaborations among organizations; 2) support local adaptation efforts; 3) mobilize support; and 4) create global public goods. We provide additional details about both methods and findings in the following three articles in this series [20-22].

### Strengths and weaknesses of the multi-method study

The multi-method study has six main strengths: 1) we examined the views and experiences of those familiar with three types of organizations that support evidence-informed policymaking, not just one of the two types of organizations previously studied (*i.e.*, we surveyed GSUs as well as CPG- and HTA-producing organizations, we interviewed roughly equal numbers of CPG- and HTA-producing organizations and GSUs, and the majority of case descriptions were GSUs); 2) we achieved both breadth (through a survey) and depth (through interviews with directors and then case descriptions that drew both on interviews with a range of staff, advocates and critics and on documentary analyses) in our examination of their views and experiences; 3) we drew on a regionally diverse project reference group to ensure that our draft protocol, study population, questionnaire, interview guide, and case description data collection protocol were fit for purpose; 4) we adapted a widely used questionnaire and achieved a high response rate with our survey (86%); 5) we used explicit sampling criteria to identify particularly successful or innovative groups for more in-depth study through interviews and case descriptions, no organization declined to participate in the interviews, and only one individual declined to participate in the interviews conducted as part of the site visits; and 6) we employed a variety of independent checks on the credibility of our thematic analyses of the written questionnaire responses and the interview and case descriptions data. The study has two main weaknesses: 1) despite significant efforts to identify organizations in LMICs, just over half (54%) of the organizations we surveyed, and just under half (48%) of the organizations we interviewed, were drawn from high-income countries; and 2) despite efforts to ask questions in neutral ways, many organizations may have been motivated by a desire to tell us what they thought we wanted to hear (*i.e.*, there may be a social desirability bias in their responses).

### What the multi-method study adds

This synthesis of findings from the first multi-method study of its kind, along with the more detailed findings from each of the three phases of the study [20-22], provides a strong basis on which researchers, policymakers and international organizations and networks can respond to the growing chorus of voices calling for efforts to support the use of research evidence in developing health policy, particularly in LMICs but also more generally. The recommendations are firmly rooted in the experiences of a remarkably diverse array of organizations, many of which are in some way successful or innovative in supporting the use of research evidence in the development of CPGs, HTAs, and health policy.

### Implications for policymakers and for international organizations and networks

Policymakers can play a strong supporting role for these organizations, both by building strong links with the organizations while respecting their independence and by encouraging them to follow the recommendations that emerged from the study, such as to collaborate with other organizations, manage conflicts of interest, build capacity, and use good methods and be transparent in the work. International organizations and networks also have a key role to play in supporting collaborations among organizations, supporting local adaptation efforts, mobilizing support, and creating global public goods. Such activities could be undertaken through international research projects and capacity-building initiatives focused on those organizations with significant but as yet unrealized potential and through educational and networking workshops that bring together researchers, policymakers, and stakeholders linked to organizations at various stages in their development (and ideally from several different countries).

### Implications for future research

A similar assessment should be repeated in a few years, by which time many new and promising organizational forms for supporting the use of research evidence in the development of health policy, such as the WHO-sponsored Evidence-Informed Policy Networks, will have been established [23]. These new organizational forms should also be evaluated prospectively to identify what works well in what contexts and why. Moreover, additional research is needed to develop and evaluate tools that these organizations can use to support the use of research evidence in developing health policy, as is being planned by the European Union-funded project, entitled Supporting the Use of Research Evidence (SURE) in African Health Systems.

## Competing interests

The authors declare that they have no financial competing interests. The study is part of a broader suite of projects undertaken to support the work of WHO Advisory Committee on Health Research (ACHR). Both JL and AO are members of the ACHR. JL is also President of the ACHR for the Pan American Health Organization (WHO's regional office for the Americas). The Chair of the WHO ACHR, a member of the PAHO ACHR, and several WHO staff members were members of the project reference group and, as such, played an advisory role in study design. Two of these individuals provided feedback on the penultimate draft of the report on which the article is based. The authors had complete independence, however, in all final decisions about study design, in data collection, analysis and interpretation, in writing and revising the article, and in the decision to submit the manuscript for publication.

## Authors' contributions

JL participated in the design of the three-phase study, participated in analyzing the qualitative data and deciding how to present the quantitative data, and drafted the article and the report in which it is based. AO conceived of the study, led its design and coordination, participated in analyzing the qualitative data, and contributed to drafting the article. RM participated in the design of the study, led the data collection for the second and third phases of the study, led the analysis of the qualitative data, and contributed to drafting the article. EP led the data collection for the first phase of the study, contributed to data collection for the other two phases, and led the analysis of the quantitative data. All authors read and approved the final manuscript.
